# Dietary Fiber Intake Improves Osteoporosis Caused by Chronic Lead Exposure by Restoring the Gut–Bone Axis

**DOI:** 10.3390/nu17091513

**Published:** 2025-04-29

**Authors:** Ruijian Wang, Jin Shen, Chunqing Han, Xiaodong Shi, Yan Gong, Xiping Hu, Zhongtang Jia, Miaomiao Wang, Yu Wu

**Affiliations:** 1 MOE Medical Basic Research Innovation Center for Gut Microbiota and Chronic Diseases, Wuxi School of Medicine, Jiangnan University, Wuxi 214126, China; wrj4610@163.com (R.W.); lmob26@163.com (J.S.); hchunqing98@163.com (C.H.); hxiping98@163.com (X.H.); jiazhongtang2021@163.com (Z.J.); 2 Laboratory of Modern Environmental Toxicology, Wuxi School of Medicine, Jiangnan University, Wuxi 214126, China; 3 Public Health Research Center, Jiangnan University, Wuxi 214064, China; 4 Department of Management Engineering, Capital University of Economics and Business, Fengtai, Beijing 100070, China; xiaoxdxd@163.com; 5 Department of Occupational Medicine, Wuxi Center for Disease Control and Prevention, Wuxi 214101, China; 15951514248@163.com

**Keywords:** dietary fiber, gut–bone axis, lead exposure, NHANES, osteoporosis

## Abstract

**Background:** Lead (Pb), a pervasive environmental toxicant with specific toxicity to bone, has been recognized as a significant etiological factor in the pathogenesis of osteoporosis. While dietary fiber (DF) demonstrates anti-osteoporotic potential, its protective role against Pb-induced bone loss remains unexplored. **Methods:** This study analyzed the association between dietary fiber, blood lead, and osteoporosis based on the NHANES database, and validated it by constructing a lead exposed mouse model. Micro CT was used to evaluate bone microstructure, ELISA was used to detect bone markers, q-PCR/Western blot was used to measure intestinal tight junction protein, flow cytometry was used to analyze Treg cells in colon/bone tissue, GC-MS was used to detect short chain fatty acids, and 16S rRNA sequencing was used to analyze changes in gut microbiota. The regulatory mechanism of dietary fiber on bone metabolism and intestinal barrier in lead exposed mice was systematically evaluated. **Results:** Based on NHANES data analysis, it was found that dietary fiber can reduce the risk of osteoporosis in lead exposed populations. Animal experiments have shown that dietary fiber intervention significantly increases bone density, improves bone microstructure and metabolic indicators, repairs intestinal barrier damage caused by lead exposure, and regulates immune balance in lead exposed mice. At the same time, it promotes the generation of short chain fatty acids and the proliferation of beneficial gut microbiota. **Conclusions:** These findings indicate that DF mitigates Pb-induced osteoporosis through gut barrier restoration, SCFA-mediated immunomodulation, and microbiota-driven Treg cell expansion along the gut–bone axis.

## 1. Introduction

Osteoporosis, a pathological condition classified among metabolic bone disorders, is characterized by four key features: reduced bone density, compromised structural quality, deterioration of microarchitectural integrity, and elevated skeletal fragility leading to heightened fracture susceptibility. It has become an increasingly serious global health problem [1]. As age increases, the incidence of osteoporosis continues to rise [2]. The incidence of osteoporosis in women is significantly higher than that in men, mainly due to the decrease in bone mineral density (BMD) and accelerated bone loss caused by estrogen deficiency in postmenopausal women [3], which makes them more prone to osteoporosis. Substantial scientific evidence indicates that environmental determinants, notably heavy metal exposure, atmospheric contaminants, and endocrine-disrupting chemicals (EDCs), are mechanistically implicated in exacerbating osteoporotic progression through interference with bone remodeling homeostasis [4].

A cross-sectional study based on NHANES found a potential association between lead exposure and osteoporosis [5,6], but further experimental verification is needed to determine whether there is a causal relationship. There are studies indicating that lead can damage the endocrine regulatory system of bone mineral balance and bone growth, resulting in negative effects on bones [7,8]. Long-term lead exposure can increase pro-inflammatory factors (such as TNF-α and IL-17), indirectly stimulate osteoclastogenesis, lead to excessive bone resorption, and reduce bone density [9].

The intestine is the main pathway for lead absorption during non-occupational lead exposure, with an absorption rate of approximately 5% to 10% in adults [10]. Epidemiological evidence demonstrates that chronic lead exposure is associated with compromised intestinal barrier integrity and concomitant dysbiotic alterations in gut microbiota (GM) homeostasis [11]. Lead can disrupt the structure of the intestinal epithelium, leading to the decreased expression of tight junction (TJ) proteins such as ZO-1 and Occludin in colon tissue. This compromises the intestinal barrier and increases its permeability [12,13]. Lead can also alter the structural configuration and ecological richness of the GM, affecting the balance between harmful and beneficial bacteria [14,15,16,17]. Many clinical studies have shown that gut microbiota can regulate bone metabolism [18,19,20]. Furthermore, distinct GM patterns have been identified in osteoporosis patients, suggesting their potential utility as biomarkers or therapeutic targets.

The regulatory mechanism through which the GM modulates bone remodeling operates via both direct and indirect pathways. Specifically, the GM directly affects bone remodeling processes through secondary microbial metabolites, including but not limited to short-chain fatty acids and amino acids, which act as signal molecules to modulate genes related to osteoclast (OC) and osteoblasts (OBs). Research has shown that SCFAs, especially butyrate and propionate, can increase bone density. They achieve this by affecting the activity of OC and OB, thereby promoting bone formation and reducing bone resorption [21]. Indirectly, the GM regulates bone remodeling by interacting with immune cells, particularly T cells and B cells, exerting immunomodulatory functions crucial for maintaining optimal bone health. Research indicates that disruptions in the gut microbiota’s balance and functionality can contribute to immune system dysregulation through processes such as the production of inflammatory metabolites, heightened intestinal permeability, and compromised regulatory T-cell activity. This cascade triggers localized and systemic inflammation via the activation of pivotal pro-inflammatory cytokines, concurrently aggravating osseous tissue destruction [22].

A study showed that dietary fiber contributed meaningfully to the composition, diversity, and richness of the gut microbiota. Dietary fiber engages in direct crosstalk with intestinal microbiota, thereby driving the biosynthesis of pivotal metabolites, including short-chain fatty acids (SCFAs) [23]. Other studies have found that SCFAs produced by gut microbiota-mediated fiber catabolism are beneficial for osseous integrity [24,25,26,27]. However, whether dietary fiber improves osteoporosis induced by lead is not yet clear. Therefore, the main objective of this article is to further elucidate the mechanism by which lead aggravates osteoporosis and specifically explore the improvement effect of dietary fiber on osteoporosis.

## 2. Materials and Methods

### 2.1. Analysis of NHANES Database

**Figure 1:**
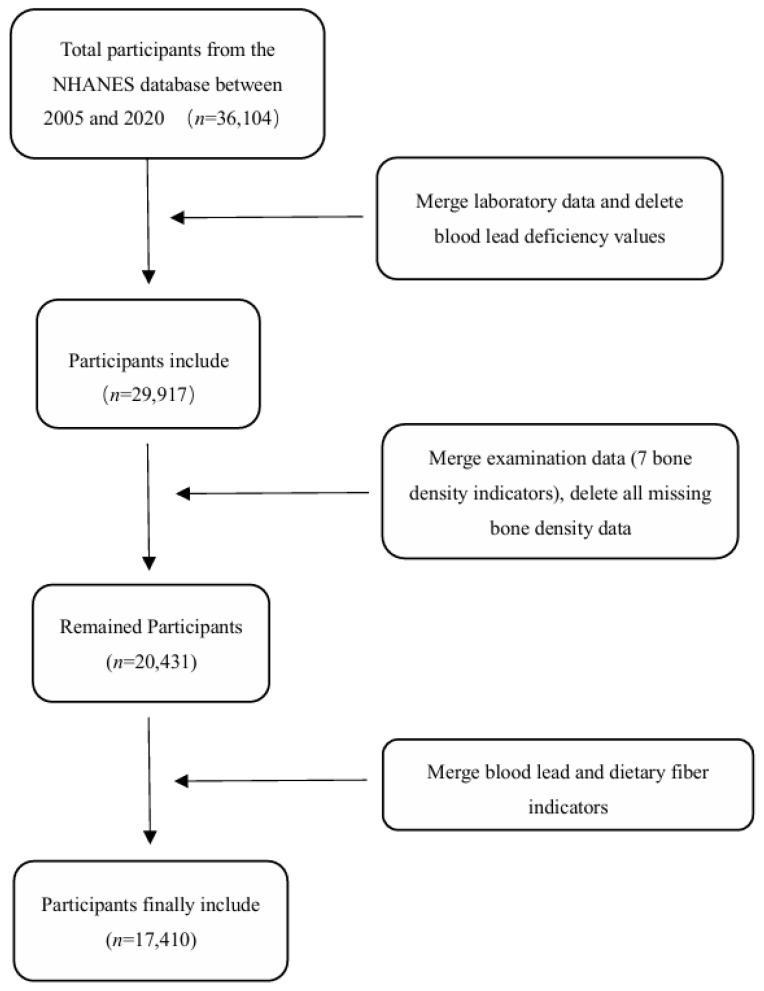
The screening process for the participants.

We analyzed NHANES data from participants aged ≥ 30 years (*n* = 36,104) collected between 2005 and 2020. After excluding individuals lacking blood lead measurements (*n* = 6187) and BMD records (*n* = 9486), 17,410 eligible participants with complete dietary fiber data were included in this cross-sectional study. Figure 1 presents a flowchart of the participant selection process. Blood lead levels were quantified at mobile examination centers using inductively coupled plasma mass spectrometry (ICP-MS) following standardized NHANES protocols. Dietary fiber intake was determined through 24 h recall surveys, with dual measurements averaged for analysis; single measurements were retained when available. Participants were stratified into low-/high-exposure groups using median splits for both blood lead and dietary fiber intake. All laboratory procedures adhered to CDC Environmental Health Laboratory quality assurance standards. Dual-energy X-ray absorptiometry (DXA) was used to measure the femoral, lumbar, and femoral neck BMD. Following the WHO criteria, osteoporosis was defined as a T-score ≤ −2.5. T-scores were calculated as follows: T-score = (Individual BMD-Reference population mean BMD)/Reference population SD. Detailed calculation methods are described elsewhere [28].

### 2.2. Animal Experiment Design

Five-week-old male C57BL/6J mice (Model Animal Research Institute, Nanjing, China) underwent 7-day acclimatization in quarantine facilities. Using a single-blind randomized design, mice (*n* = 10/group) were allocated to one of three groups: (1) The control group received physiological saline (free drinking) + 0% dietary fiber diet. (2) The lead exposure group received 50 ppm lead acetate solution (free drinking) + 0% dietary fiber diet. (3) The dietary fiber intervention group received 50 ppm lead acetate solution (free drinking) + 10% dietary fiber diet.

The treatments were administered daily for 4 months. During the exposure period, we weighed the mice every two weeks and observed their behavior to determine if their condition was good. The data of mice with diseases or deaths unrelated to the experiment were excluded. The final sample size was 6–10 per group. When conducting Western blot experiments, because it is consistent with the results of qPCR, we used three independent samples instead of technical replicates of the same animal. Blood was collected following anesthesia with an intraperitoneal injection of pentobarbital sodium (50 mg/kg, 0.1 mL), and euthanasia was carried out through cervical dislocation for sampling. All procedures strictly complied with the ARRIVE guidelines and were approved by the Ethics Committee of Jiangnan University (JN. No.20220315c0900815 [075]).

### 2.3. Tissue Harvesting and Processing

Before euthanizing the mice, they were placed in a disposable feeding box and left to defecate naturally. A total of 5–6 pieces of feces were collected from each mouse, taking care to avoid contamination by urine. Part of the collected feces was freeze-dried at a low temperature to detect the short-chain fatty acid content, while the other part was stored at −80 °C for analysis of the gut microbiota. Following 12 h of fasting, the mice were anesthetized, and their whiskers were removed. One eyeball was removed quickly with sterilized forceps, and blood samples were collected. The serum was separated using centrifugation at 4000× *g* for 10 min (4 °C) and stored at −80 °C. The right femur was dissected, fixed in 4% paraformaldehyde (PFA), and preserved for micro-CT analysis. The contralateral leg bones were snap-frozen in 1.5 mL microcentrifuge tubes at −80 °C for molecular analyses. A 2 cm distal colonic segment was excised below the cecum. After PBS perfusion, fresh specimens underwent flow cytometry analysis. Tissue aliquots were either flash-frozen (−80 °C) for mRNA/protein studies or PFA-fixed for histological examination.

### 2.4. Lead Quantification

Serum lead levels were determined using atomic absorption spectroscopy (AAS; PinAAcle 900Z, PerkinElmer, Waltham, MA, USA) following nitric acid digestion (15% *v*/*v* in bovine serum), with inter- and intra-assay CVs < 10%. Absorbance measurements at 283.3 nm were calibrated against NIST-traceable standards. All procedures included triplicate measurements and blank controls.

### 2.5. Micro-CT Analysis

Mouse femurs were pre-fixed with 4% paraformaldehyde (PFA) for 24 h and then stored in 70% ethanol. Fixed femoral specimens were scanned using a Bruker Skyscan 1276 system at 60 kV, 200 μA with an isotropic resolution of 9 μm. A 0.5 mm aluminum (Al) filter was applied, and a 0.4° step size was used. A 1.25 mm trabecular bone region, starting 0.3 mm proximal to the growth plate, was analyzed using CTAn software (v1.20.8.0) to quantify the following parameters: bone volume fraction (BV/TV), trabecular number (Tb.N), trabecular thickness (Tb.Th), trabecular separation (Tb.Sp), and bone surface density (BS/TV).

### 2.6. ELISA

Serum concentrations of bone turnover markers, including propeptide of type I procollagen (PINP) and C-terminal telopeptide of type 1 collagen (CTX), were determined using commercial ELISA kits (MLBio, Shanghai, China; PINP: Cat#038554, CTX: Cat#060531). Absorbance at 450 nm was measured on a BioTek Synergy H1 microplate reader. The detection range of PINP is between 2.5 and 80 ng/mL, while that of CTX is between 0.5 and 50 ng/mL, with inter- and intra-assay CVs of <8%. All samples were analyzed in triplicate.

### 2.7. Hematoxylin and Eosin (H&E) Staining

According to the Scientific Compass Service platform protocol, colonic segments were fixed in 4% neutral-buffered formalin (48 h), processed through graded ethanol series, and paraffin-embedded. Tissue blocks were sectioned at 4 μm thicknesses using a Leica RM2235 microtome (Leica Biosystems, Munic, Germany). After deparaffinization, H&E staining was performed using a kit C0105 (Beyotime, Shanghai, China) following the manufacturer’s protocols. Digital images were acquired with a 3DHISTECH Pannoramic MIDI scanner (Budapest, Hungary) and analyzed using CaseViewer software (v2.4). Pathological sections of the colon tissue were observed to evaluate the tissue structure and determine whether inflammatory cells and mucosal damage occurred.

Scoring was based on the evaluation method described in the literature [29]. Histopathological evaluation was conducted using the following grading criteria: crypt damage (scored on a 0–4 scale), inflammatory severity (assessed with a 0–3 grading system), and goblet cell impairment (evaluated using a 0–3 scoring range). For quantitative analysis of mucus-secreting goblet cells within intestinal crypts, Image-Pro Plus (6.0) image analysis software (Media Cybernetics, Inc., Rockville, MD, USA) was employed to perform the microscopic observation.

### 2.8. RNA Extraction and qRT-PCR Analysis

Colonic RNA was isolated using TRIzol reagent (Takara, Shiga, Japan). The RNA concentration and purity were measured by UV spectrophotometry (NanoDrop^®^ ND-2000, Thermo Fisher Scientific, Waltham, MA, USA). cDNA synthesis was performed with a PrimeScript™ RT Reagent Kit (RR047B, Takara, San Jose, CA, USA). Quantitative reverse transcription PCR (qRT-PCR) was conducted on an Applied Biosystems 7500 Real-Time PCR System using SYBR Premix Ex Taq™ (RR820A, Takara). Target genes were amplified with specific forward and reverse primer pairs (Table 1), synthesized by Sangon Biotech (Shanghai, China). The relative expression of target genes were calculated by ΔΔCT: (1) Experimental group CT value—Control group CT value = ΔCT; (2) Δct value of experimental group—Δ ct value of control group = ΔΔCT; (3) use the function power (2, −ΔΔCT) to obtain specific values, and then use GraphPad Prism 9.4.1 for visualization analysis.

### 2.9. Extraction of Bone Tissue Protein

The bone tissue was removed and stored in a −80 °C freezer. Then, any soft tissue attached to the surface of the bone tissue was removed and rinsed thoroughly with PBS. The mortar was pre-cooled, and the washed bone tissue was frozen with liquid nitrogen. Then, it was ground into powder and collected in an EP tube. Subsequently, RIPA lysis buffer containing protease inhibitors was added to an EP tube containing bone tissue powder, and the mixture was lysed for 15 min. The sample was then centrifuged at 15,000 rpm for 15 min, and the supernatant was collected to determine the protein concentration.

### 2.10. Western Blot Analysis

Total proteins were isolated from the colon and bone tissues with RIPA lysis buffer (Beyotime, Shanghai, China) supplemented with protease inhibitors. The protein levels were measured using the BCA method (Thermo Fisher Scientific). The samples underwent electrophoretic separation through 10% SDS-PAGE gels followed by electroblotting onto PVDF membranes (Millipore, Burlington MA, USA). After blocking with 5% non-fat milk at room temperature for 60 min, the membranes were exposed to primary antibodies for overnight incubation at 4 °C. Following thorough washing, HRP-linked secondary antibodies (Abcam, Milpitas, CA, USA; 1:10,000 dilution) were applied for 60 min of incubation at 37 °C. Detection was performed using an ECL system (Tanon, Shanghai, China), and band intensity quantification was conducted with ImageJ analysis software 6.0 (NIH, MD, USA). The primary antibodies involved included ZO-1 (MCE, NJ, USA, dilution ratio of 1:1000), Occludin (MCE, NJ, USA, dilution ratio of 1:1000), Runx2 (Proteintech, Wuhan, China, dilution ratio of 1:3000), Sp7 (ABclonal, Wuhan, China, dilution ratio of 1:800), and GAPDH (Proteintech, Wuhan, China, dilution ratio of 1:10,000).

### 2.11. Flow Cytometry for Treg Cell Analysis

The mouse intestine was dissected, and excess adipose tissue was removed. The contents of the intestine were washed with PBS. Then, a glass rod of appropriate thickness was inserted into the intestine to excise the Peyer’s patches (oval-shaped milky white or light pink protrusions) [30]. Due to the small number of Peyer patches in a single mouse, it does not meet the requirements for machine testing. Therefore, two mice in the same group were combined into an independent sample for testing. Bone marrow cells were flushed from the femurs, followed by red blood cell lysis (Elabscience, Houston, TX, USA, E-CK-A109). The cell suspensions were adjusted to 1 × 10^7^ cells/mL and stained according to the mouse Treg Cell Kit protocol (Elabscience, E-CK-A109): (1) Fc receptor blocking with anti-CD16/32 antibody (E-AB-F0997A) for 10 min; (2) surface staining with FITC-anti-CD4 and APC-anti-CD25 (30 min, 4 °C in dark); (3) intracellular FoxP3 staining using PE-anti-FoxP3 after fixation/permeabilization (30 min, RT). The cell suspensions were prepared in 200 μL of staining buffer (E-CK-A107) and analyzed with flow cytometry using a BD FACSAria III system (BD Biosciences, Franklin Lakes, NJ, USA). Subsequent data analysis was conducted utilizing FlowJo software (version 10.8.1).

### 2.12. Quantification of Short-Chain Fatty Acids

SCFAs in the mouse feces were quantified through GC-MS following established protocols [28]. The samples were processed by thoroughly mixing with diethyl ether containing 15% phosphoric acid and 75 µg/mL isocaproic acid (serving as an internal standard) and then centrifuged at 12,000× *g* for 10 min under 4 °C conditions. Subsequent analysis of the supernatant was conducted using a Thermo Scientific TRACE 1310 gas chromatography system paired with an ISQ 7000 mass spectrometer (Thermo Fisher Scientific, Dreieich, Germany).

Separation was accomplished using an Rtx-Wax capillary column (30 m length × 0.25 mm internal diameter, 0.25 µm stationary phase thickness; Restek, Centre County, PA, USA) with helium maintained at a 1.4 mL/min flow rate. The injection port was operated at 240 °C under split mode (10:1 ratio) with a 1 µL sample introduction. The thermal gradient protocol comprised 2 min of stabilization at 100 °C, heating to 140 °C at 7.5 °C/min, subsequent rapid elevation to 200 °C at 60 °C/min, and 3 min of isothermal maintenance. Mass spectrometric detection was performed using an electron impact ionization (70 eV) with a 220 °C ion source. Quantitative determination of short-chain fatty acids (propionic, butyric, and valeric acids) was executed through Xcalibur 4.3 analytical software (Thermo Fisher Scientific) utilizing pre-established calibration curves.

### 2.13. 16S rRNA Gene Sequencing

The mouse feces stored at −80 °C were retrieved, and microbial genomic DNA was extracted using the QIAamp DNA Stool Mini Kit (Qiagen, Hilden, Germany). The V3-V4 hypervariable regions of bacterial 16S rRNA genes were amplified and sequenced on an Illumina HiSeq 2500 platform (2 × 250 bp paired-end reads; Illumina, San Diego, CA, USA). The reference database used was National Center for Biotechnology Information (NCBI).

Bioinformatics analysis included the following: OTU clustering: Sequences were processed using QIIME2 (v2023.2) and clustered into operational taxonomic units (OTUs) at 97% similarity with VSEARCH. Diversity analysis: The α-diversity (Shannon index, Chao1) was calculated using the observed OTUs. The β-diversity was assessed via non-metric multidimensional scaling (NMDS) based on Bray–Curtis distances. LEfSe analysis: Linear discriminant analysis effect size (LDA score ≥ 2.0, Kruskal–Wallis *p* < 0.05) was used to identify differentially abundant taxa. Correlation analysis: Spearman’s rank correlations between microbial taxa and SCFAs were computed in R (v4.3.1) using the vegan package.

### 2.14. Statistical Analysis

The experimental results are displayed as the arithmetic mean values with the standard deviation (SD) ranges. The normality of distribution was verified using the Shapiro–Wilk statistical procedure, while the homogeneity of variance was evaluated using Levene’s analytical method. For NHANES dataset analysis and experimental data analysis, the following tests were performed.

For normally distributed data, Student’s *t*-test and one-way ANOVA were applied; for non-normal/homogeneous variance data, the Mann–Whitney U test and Kruskal–Wallis test were carried out. Statistical significance was defined as *p* < 0.05. All analyses were performed using GraphPad Prism 9.4.1 (GraphPad Software, San Diego, CA, USA).

## 3. Results

### 3.1. Dietary Fiber Intake Is Negatively Correlated Osteoporosis in Lead Exposure Population with NHANES

At the population level, we selected 36,104 participants aged 30 and above from the NHANES database, excluding those with missing blood lead and bone density data. In total, we included 17,410 participants, as detailed in Figure 1. A demographic analysis was conducted on the 17,410 eligible participants, and it was found that in the population with osteoporosis, blood lead levels were higher than those in the non-osteoporosis group, and dietary fiber intake was lower than that in the non-osteoporosis group (Table 2). To investigate the association between blood lead levels, dietary fiber intake, and osteoporosis, we conducted a correlation analysis and found that lead exposure is a risk factor for osteoporosis, while dietary fiber intake is a protective factor for osteoporosis (Table 3). Therefore, we further evaluated the impact of dietary fiber on the development of osteoporosis in a population with different lead burdens, using the category “low lead and high dietary fiber intake” as the reference. Multivariate logistic regression modeling demonstrated that cumulative lead exposure elevation and reduced dietary fiber intake were dose-dependently associated with elevated osteoporosis risk (Table 4).

### 3.2. DF Improved Bone Microstructure Damage in Pb-Exposed Mice

**Figure 2:**
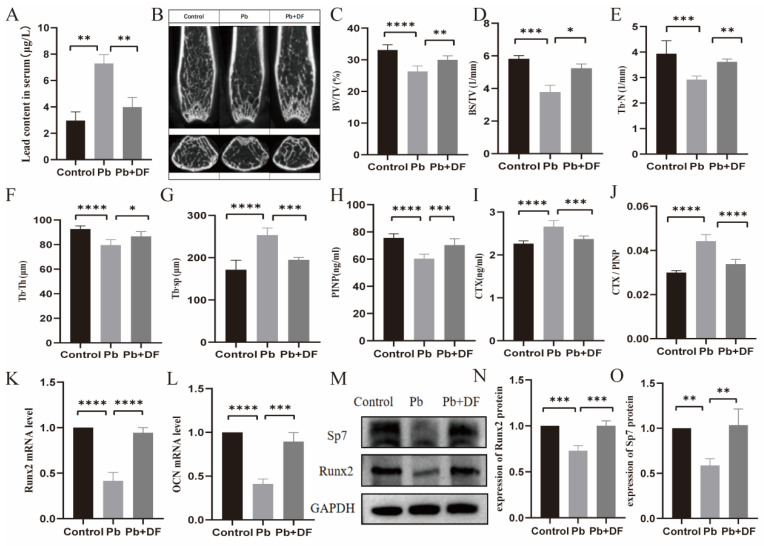
Dietary fiber can improve osteoporosis in lead-exposed mice. (**A**) Lead content in the serum of mice in each group. (**B**) The distal metaphyseal region of murine femurs was evaluated through micro-computed tomography (micro-CT) imaging. (**C**–**G**) Comparison of BV/TV, BS/TV, Tb.N, Tb.Th, and Tb.Sp among groups. (*n* = 6–10). (**H**–**J**) Changes in the serum PINP levels, CTX-I, and CTX-I/PINP were observed among the groups (*n* = 6–10). (**K**,**L**) The mRNA expression of the Runx2 and OCN gene in bone tissue (*n* = 6–10). (**M**–**O**) Western blot assay revealed changes in Sp7 and Runx2 protein expression (*n* = 3). Data are expressed as the mean ± standard deviation. * *p* < 0.05, ** *p* < 0.01, *** *p* < 0.001, and **** *p* < 0.0001 (Dunnett multiple-comparison test).

To investigate the effect of dietary fiber on lead-induced bone loss, we established chronic lead exposure mouse models and evaluated the effect of dietary fiber intake. The initial quantification of murine blood lead levels revealed significantly elevated serum lead concentrations in the lead-exposed group compared with the controls, whereas dietary fiber supplementation resulted in a marked reduction relative to lead-exposed animals. This proves the successful construction of the mouse model (Figure 2A). We further evaluated the effects of lead exposure-induced bone loss using micro-computed tomography (μ-CT) (Figure 2B). Compared with the control group, the Pb-exposed group had thinner, fewer, and sparsely arranged distal femoral trabeculae and larger bone marrow cavities. However, the femur horizontal cross-section of the chronic lead-exposed mice in the dietary fiber intervention group showed an increase in bone density and an improvement in the microstructure of bone trabeculae, as reflected by the enhancement in the bone volume/total volume (BV/TV), bone surface area/total volume (BS/TV), trabecular number (Tb.N), and trabecular thickness (Tb.Th), and the reduction of trabecular separation (Tb.Sp) (Figure 2C–G). Consistently, supplementing with dietary fiber significantly increased the serum PINP levels and decreased the CTX expression levels compared with the lead-exposed mice (Figure 2H–J). Moreover, decreased expressions of Runt-related transcription factor 2 (Runx2) and Osteocalcin (OCN), two markers for bone formation, were found in the Pb exposure group, which increased after dietary fiber intake (Figure 2K,L). Similarly, the protein expression of Runx2 and Sp7 also increased with the DF treatment (Figure 2M–O).

### 3.3. DF Repaired the Intestinal Barrier Damaged by Pb

**Figure 3:**
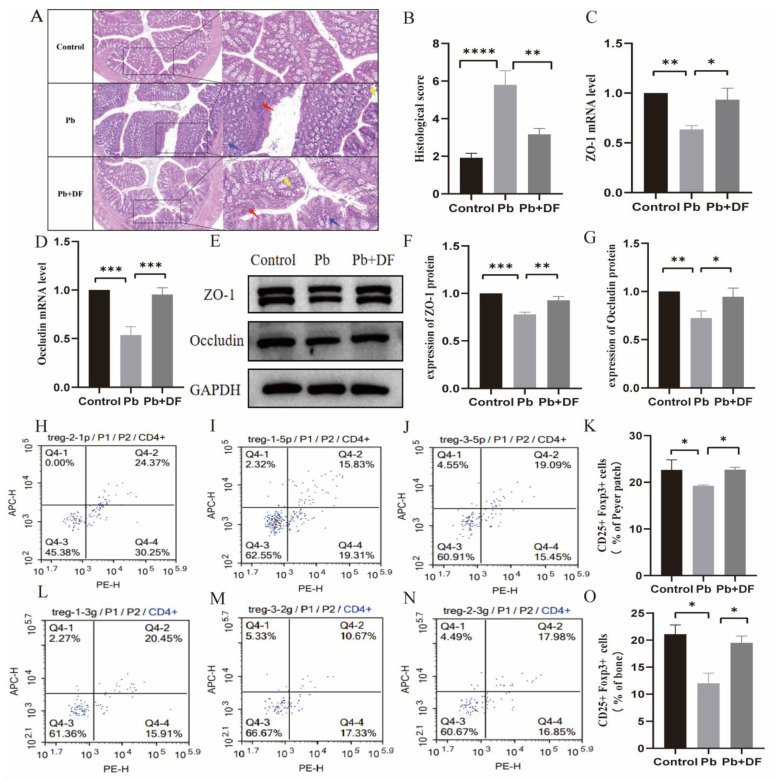
DF restored intestinal structure and Treg cells disrupted by Pb exposure. (**A**) Hematoxylin and eosin-stained sections (*n* = 6–10) at a 200 µm scale, with insets showing 100 µm-magnified regions. Annotations: inflammatory infiltrates (red arrows), goblet cells (blue arrows), intestinal crypts (yellow arrows). (**B**) Studies on intestinal morphology involved the assessment of cumulative scores encompassing inflammatory infiltration, crypt damage, and goblet cell impairment. (**C**,**D**) The mRNA expression of the ZO-1 and Occludin gene in colon tissue (*n* = 6–10). (**E**–**G**) The protein expression of the ZO-1 and Occludin gene in colon tissue (*n* = 3). (**H**–**J**) Representative flow cytometry image of Treg cell subpopulations in the colon. (**K**) Percentage of Treg cell subsets in CD4 ^+^ T cells in mice colon (*n* = 6–10). (**L**–**N**) Representative flow cytometry images of Treg cell subpopulations in the bone. (**O**) Percentage of Treg cell subsets in CD4 + T cells in mice bone (*n* = 6–10). Data are presented as mean ± SD. * *p* < 0.05, ** *p*< 0.01, *** *p* < 0.001, and **** *p* < 0.0001 (Dunnett multiple-comparison test).

The increase in intestinal permeability plays a key role in the upregulation of the inflammatory response. To investigate the possible mechanism regarding the protection effect of DF on Pb-induced bone loss, we first evaluated the integrity of the intestinal barrier. Representative images from H&E staining of the colon structure show that lead exposure led to local damage to tissues and mucous membranes, atrophy and deformation of intestinal glands, and partial replacement by proliferating fibrous tissue, and infiltration of inflammatory cells (Figure 3A,B). Taking dietary fiber can reduce mucosal damage and inflammatory cell infiltration caused by lead exposure and improve crypt structure (Figure 3A,B). In addition, we found that after taking dietary fiber, the mRNA and protein expression levels of TJ protein ZO-1 and Occludin were significantly increased (Figure 3C–G). We further examined the expression of T-cell subsets (Treg) in the Peyer patches and femur bone marrow. Compared with the control group, the Treg levels in the lead-exposed group were significantly reduced in the colon, while after dietary fiber supplementation, the Treg levels in both the colon and bone increased significantly (Figure 3H–O).

### 3.4. DF Regulated Changes in Gut Microbiota Structure in Mice Exposed to Pb

**Figure 4:**
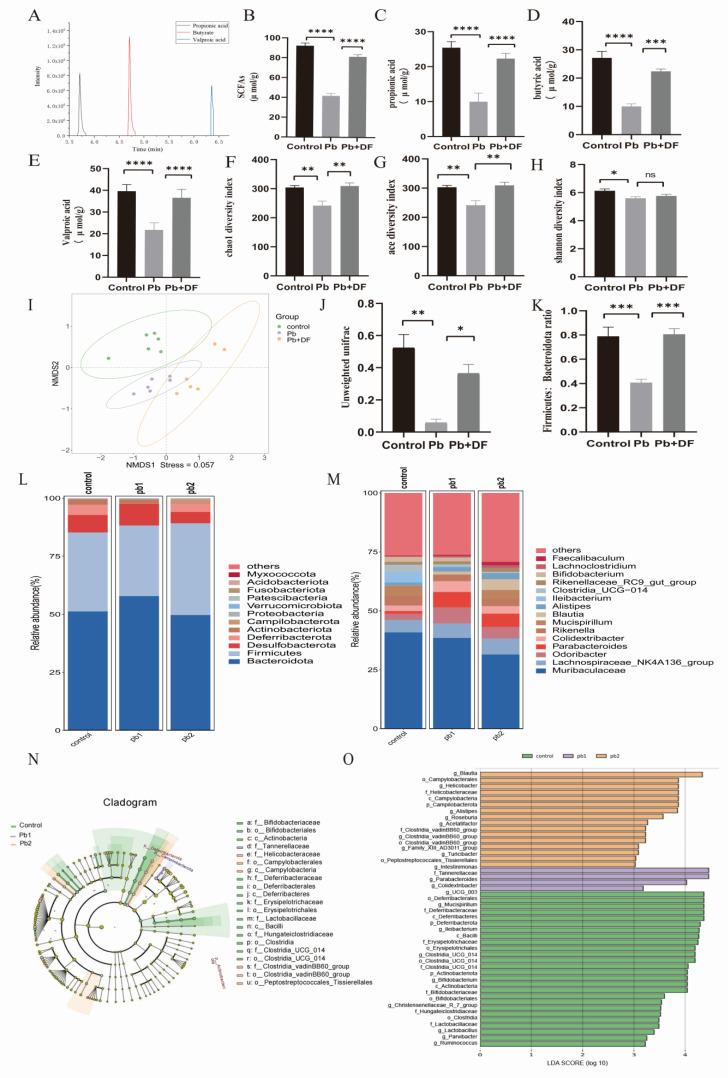
DF intervention upregulated SCFAs levels and improved gut microbiota composition in lead-exposed mice. (**A**) Standard curve chromatogram of SCFAs. (**B**–**E**) Expression levels of SCFAs in mouse feces (*n* = 6–10). (**F**–**H**) Species richness and species diversity index. (**I**) NMDS analysis based on unweighted UniFrac distances. (**J**) Unweighted UniFrac distances. (**L**,**M**) Comparative profiles of microbial abundance at the phylum (**left** panel) and class (**right** panel) taxonomic tiers across the designated experimental cohorts (*n* = 6–10). (**K**) Firmicutes: Bacteroidetes proportion. (**N**,**O**) LEfSe (linear discriminant analysis effect size) delineating discriminatory taxa with differential abundance profiles in microbial community enrichment analyses. pb1: Lead exposure group. pb2: Lead exposure and dietary fiber intervention. Data are presented as mean ± SD. * *p* < 0.05, ** *p* < 0.01, *** *p* < 0.001, and **** *p* < 0.0001. ns, *p >* 0.05 (Dunnett multiple-comparison test).

Through GC-MS analysis of mouse feces, it was found that Pb exposure led to a decrease in the content of SCFAs in the mouse body, while DF intake compensated for the deficiency of SCFAs caused by Pb exposure (Figure 4A–E). Since changes in SCFAs are affected by the GM, we further examined the changes in the gut microbiota composition. Regarding the alpha diversity of the GM, lead exposure reduced species richness and diversity in the gut, as reflected by the decreases in the Chao 1 index, ACE index, and Shannon index. After supplementing dietary fiber, there was a significant increase in species richness, while species diversity showed an upward trend, but there was no significant difference (Figure 4F–H). NMDS analysis based on non-metric multidimensional scaling and unweighted UniFrac distances showed significant and distinct clustering of the gut microbiota among the groups (NMDS1 = 0.057; Figure 4I,J). A decreased expression of Deferribacterota and Bacteroidota in the intestine and an increased expression of Desulfobacterota were found in the lead-exposed mice. Dietary fiber intervention can upregulate the levels of Deferribacterota and Bacteroidota in lead-exposed mice and downregulate Desulfobacterota (Figure 4L). Further analysis at the genus level revealed a decrease in the expression levels of Mucispirillum and Rikenella in lead-exposed mice. The functions of Mucusoirillum and Rikenella are mainly reflected in enhancing the intestinal barrier, affecting metabolic balance, and promoting intestinal health. However, taking dietary fiber can upregulate the expression levels of Mucusoirillum and Rikenella in lead-exposed mice (Figure 4M). Lead exposure increased the Firmicutes/Bacteroidetes (F/B) ratio, while dietary fiber intervention reversed this trend (Figure 4K). LEfSe analysis (analysis score > 3) and the LDA branching plot showed that compared with the control group, the lead exposure group showed a significant decrease in the proportion of dominant bacterial strains. However, compared with the lead exposure group, the proportion of dominant bacterial strains significantly increased in the dietary fiber intervention group. Only two dominant bacteria related to maintaining intestinal health, Parabacterioids and UGG, were involved in the lead exposure group. In the dietary fiber intervention group, the proportion of dominant bacterial strains significantly increased. Common strains involved in preventing colitis and maintaining intestinal microbiota balance include Mucispirillum, Bifidobacterium, Lactobacillus, and Ruminococcus (Figure 4N,O).

## 4. Discussion

Bone injury is a harmful biological effect of heavy metal lead, but the potential mechanism of lead-induced bone toxicity is still unclear, which hinders the prevention and management of bone-related diseases caused by lead exposure. Although dietary fiber has been proven to improve bones, there is currently no evidence to support its protective effect on osteoporosis caused by lead exposure. Recently, we found that restoring the gut–bone axis helps maintain bone integrity in lead-exposed mice [28]. Here, we further reveal that supplementing dietary fiber can improve osteoporosis caused by chronic lead exposure.

We first explored this issue using population databases and found a protective effect of DF on the lead burden population. Dietary patterns were associated with the risk of osteoporosis and fractures [31]. One study based on the NHANES database also showed that dietary patterns played a key role in osteoporosis in middle-aged and elderly populations [32]. To further investigate the effect of dietary patterns on osteoporosis related to lead exposure, we conducted a correlation analysis between blood lead exposure levels, DF intake levels, and the risk of osteoporosis based on survey data from the NHANES database. We found that among lead-exposed populations, those with higher dietary fiber intake had a lower risk of osteoporosis. We then validated the observational results of the population using a chronic lead exposure mouse model and observed an increase in the bone density of lead-exposed mice after dietary fiber intervention. Research found that after dietary fiber intervention, the bone microstructure of lead-exposed mice was also improved, as manifested in increased BV/TV, BS/TV, Tb. N, and Tb.Th, and decreased Tb. Sp. Moreover, after supplementing dietary fiber, the expression of CTX-I in the serum of the lead-exposed mice decreased, while the expression of PINP increased. These experimental results suggest that supplementing dietary fiber can help reduce bone resorption, increase bone formation, and promote osteogenic expression in lead-exposed mice. These findings from the observational and experimental studies indicate that dietary fiber intake is a promising intervention for the management of osteoporosis in lead-exposed populations.

Lead exposure can cause damage to intestinal function by inducing damage or apoptosis of intestinal epithelial cells, impairing intestinal absorption function, secretion function, and enhancing intestinal permeability. According to reports, long-term lead exposure can lead to the destruction of the GM and reduce SCFAs, such as propionic acid, butyric acid, and acetate [33,34,35,36]. Studies have also reported that during chronic lead exposure, Pb has an extremely long half-life in the bones, and during periods of high bone turnover or absorption, blood lead levels may increase, leading to a decrease in bone density [37]. Dietary fiber can improve intestinal integrity, reshape the GM, and increase levels of SCFAs [38]. Rizzoli et al. also demonstrated that dietary patterns may prevent osteoporosis through their impact on gut microbiota composition or function [39,40]. High dietary fiber can significantly improve the composition and abundance of gut microbiota, thereby alleviating osteoporosis [41]. Therefore, we speculate that dietary fiber can improve bone damage caused by lead exposure by restoring the gut–bone axis. Our research findings, consistent with previous reports [42], indicate that mice exposed to lead exhibited intestinal barrier damage. Dietary fiber supplementation ameliorated lead-induced epithelial damage and neutrophil infiltration in mice while restoring intestinal crypt architecture. Lead promotes excessive generation of reactive oxygen species (ROS) [43], damages mitochondrial function [44], damages cellular structure, and leads to mucosal damage and inflammatory infiltration. According to literature research [45,46], we found that dietary fiber activates the anti-inflammatory pathway and regulates the microbiota mediated by SCFAs. Therefore, it may promote the transcription of ZO-1 and occludin, reverse the expression of tight junction proteins, and repair mucosal integrity. Therefore, we detected the protein expression levels of ZO-1 and Occludin, as well as the expression level of mRAN, and found that the intervention significantly enhanced the expression of intentional tight junction proteins ZO-1 and Occludin. Moreover, our results indicate that dietary fiber intervention enhanced the expression of Treg cells in lead-exposed mice. Research has shown that disruption of the intestine structure can lead to inflammation, but dietary fiber reduces inflammation in the intestine [47]. Our findings suggest that dietary fiber can improve the gut barrier and relieve the inflammation status in lead-exposed mice.

In this study, animal experiments further showed that dietary fiber intervention effectively increased the expression levels of SCFAs, which were reduced in lead-exposed mice. SCFAs, as key microbial-derived metabolites, function to preserve the intestinal mucosal barrier, alleviate inflammatory responses, and sustain intestinal environmental homeostasis [48]. Our experimental results show that after dietary fiber intervention, the gut microbiota of lead-exposed mice improved. Epidemiological investigations have confirmed that the intake of DF is closely related to human health by the regulation of gut microbiota [49]. Dietary fiber enhances gut microbiota diversity, modulates the balance between beneficial and pathogenic bacteria, alleviates intestinal inflammation, and preserves intestinal barrier integrity through the production of beneficial metabolites [50]. Therefore, we explored the mechanism by which DF supplementation affects the GM of lead-exposed mice from three aspects: gut microbiota diversity, gut microbiota structure, and gut microbiota function.

Our research findings indicate that DF intervention can increase the diversity of gut microbiota in lead-exposed mice, as reflected by significant increases in the Chao1 index and ACE index, markers for gut microbiota diversity, which can improve their gut health and enhance their immunity. It is worth noting that the F/B ratio, as a key indicator of the gut microbiota structure, is believed to be associated with inflammation [51], and dietary fiber intervention can restore this ratio in lead-exposed mice. Furthermore, our study demonstrates that dietary fiber ameliorated gut microbiota dysfunction in lead-exposed mice. Emerging evidence indicates that intestinal lactobacilli not only promote the growth of beneficial commensals but also suppress pathogenic microorganism overgrowth, thereby contributing to intestinal homeostasis maintenance [52]. The Ruminococcus genus has also been demonstrated to correlate with decreased intestinal inflammation and the generation of secondary bile acids [53]. There are reports that Escherichia coli is positively correlated with the antioxidant capacity and glucose metabolism of skeletal muscle [54]. Coincidentally, in our study, we found that after dietary fiber intervention, the levels of lactobacilli and Ruminococcus in lead-exposed mice increased, while the levels of Escherichia coli decreased. This suggests that dietary fiber intervention can regulate the abundance of flora and the composition of intestinal flora to perform different functions.

Our current investigation further revealed that dietary fiber intervention substantially decreased the systemic lead burden in Pb-challenged mice. Emerging evidence highlights the critical role of dietary fiber in mitigating heavy metal toxicity. Research has shown that the combined supplementation of dietary fiber and polyphenols significantly reduces lead accumulation in blood and liver tissues of Pb-exposed mice [13], and analyses of a human cohort also showed inverse correlations between dietary fiber intake and blood lead levels [55]. We speculate that this protective effect may be attributed to two synergistic pathways: (1) Microbial modulation: Fiber fermentation enhances the proliferation of Pb-binding bacterial taxa (e.g., Lactobacillus, Bifidobacterium), which directly immobilize lead ions via cell surface adsorption. (2) Barrier restoration: The intestinal barrier function is enhanced by short-chain fatty acids (SCFAs) derived from dietary fiber, which promote the expression of tight junction proteins such as Occludin and ZO-1, thereby limiting paracellular Pb translocation [56].

In summary, our study demonstrates that dietary fiber intake reduced lead-associated osteoporosis risk at both population and murine levels. Dietary fiber intervention restored the tight junction complex of the epithelium by reshaping the composition of the gut microbiota, weakened inflammatory mediators along the gut–bone axis, and enhanced Treg-mediated immune regulation to counteract the effects of lead exposure. However, this study also has some limitations and shortcomings. Firstly, this study lacks evidence from a wider range of independent populations. Secondly, when analyzing NHANES data, we only briefly observed the association between lead and dietary fiber and osteoporosis without further controlling for the influence of confounding factors. In the prevention and treatment of osteoporosis in the future, the gut–bone axis can be a new focus. The core value of this focus lies in overcoming the limitations of traditional “local bone treatment” and providing innovative solutions from three dimensions: systemic metabolic regulation, environmental toxicity intervention, and personalized nutrition. In the future, it will be necessary to deepen mechanism analyses, such as exploring the impact of microbiota–host coevolution on bone metabolism, and to assess the validity of clinical translation to verify the effectiveness of dietary fiber intervention. The ultimate goal is to shift from “treating existing diseases” to “preventing diseases before they occur”.

## 5. Conclusions

Our population data research indicates that lead is a risk factor for osteoporosis, while dietary fiber can serve as a protective factor against osteoporosis. Experimental evidence from murine models revealed that dietary fiber supplementation mitigated lead (Pb)-induced osteoporosis, mediated via the restoration of gut–bone axis homeostasis. Chronic Pb exposure was observed to disrupt this axis through intestinal microbial dysbiosis, resulting in diminished short-chain fatty acid biosynthesis. Dietary fiber intervention can enhance the intestinal barrier of chronic lead-exposed mice, increase the abundance of Treg cells, and alleviate osteoporosis. Our study provides a new perspective on the prevention of osteoporosis in individuals with chronic lead exposure, emphasizing the benefits of dietary fiber.

## Figures and Tables

**Table 1:** Primers used in RT-PCR analysis.

	Forward (5′−3′)	Reverse (5′−3′)
ZO-1	GGGGAAACCCGAAACTGATGC	GTGGAGAGAAGAGTTGGACAGAGGC
Occludin	TACTGGTCTCTACGTGGATCAAT	TTCTTCGGGTTTTCACAGCAA
Runx2	GCTTCTCCAACCCACGAATG	GAACTGATAGGACGCTGACGA
OCN	CTGACCTCACAGATCCCAAGC	TGGTCTGATAGCTCGTCACAAG
GAPDH	CGTATCGGACGCCTGGTT	AGGTCAATGAAGGGGTCGTT

**Table 2:** Comparison of blood lead levels and dietary fiber intake between individuals with osteoporosis and those without osteoporosis.

Variables, *n* (%)	Osteoporosis	Non-Osteoporosis	*p* Value
Blood lead (mean ± SD)	1.81 ± 1.61	1.69 ± 1.66	<0.001
Dietary fiber intake	16.16 ± 8.39	16.87 ± 8.94	0.0051

**Table 3:** Associations of blood lead level and dietary fiber intake with the prevalence of osteoporosis.

Variables, *n* (%)	Osteoporosis	Non-Osteoporosis	OR (95%CI)	*p* Value
Blood lead	720 (44.64%)	8014 (50.73%)	1.28 (1.15–1.42)	<0.001
	893 (55.36%)	7783 (49.27%)		
Dietary fiber	845 (52.39%)	7867 (49.80%)	0.90 (0.81–1.00)	0.048
	768 (47.61%)	7930 (50.20%)		

**Table 4:** Subgroup analysis of associations of blood lead level and dietary fiber intake with osteoporosis.

Group	Osteoporosis	Non-Osteoporosis	OR (95%CI)	*p* Value
HL dietary fiber and LL Pb	364 (22.57%)	4130 (26.14%)	1.00 (reference)	
LL dietary fiber and LL Pb	356 (22.07%)	3884 (24.59%)	1.04	0.62
HL dietary fiber and HL Pb	404 (25.05%)	3800 (24.06%)	1.21	0.013
LL dietary fiber and HL Pb	489 (30.32%)	3983 (25.21%)	1.39	<0.001

## Data Availability

Data will be made available upon request.
